# Supplementary material for: A CT-Based Clinical, Radiological and Radiomic Machine Learning Model for Predicting Malignancy of Solid Renal Tumors (UroCCR-75)
Source: Diagnostics (Basel). 2023 Jul 31;13(15):2548. doi: 10.3390/diagnostics13152548 (PMC10417436; doi:10.3390/diagnostics13152548)
Supplement: Supplementary file 1 [file diagnostics-13-02548-s001.zip › diagnostics-2267584-supplementary.pdf]

SUPPLEMENTAL TABLE

Supplemental Table. Full list of the 35 radiomic features extracted based on Kendall's correlation coefficient.

Radiomic Feature	
Area.density…aligned.bounding.box	Morphological features
Area.density…convex.hull	Morphological features
Area.density…oriented.bounding.box	Morphological features
Centre.of.mass.shift..cm.	Morphological features
Flatness	Morphological features
Inverse.elongation	Morphological features
Spherical.disproportion	Morphological features
Volume.density…aligned.bounding.box	Morphological features
Volume.density…enclosing.ellipsoid	Morphological features
Volume.density…oriented.bounding.box	Morphological features
Number.of.grey.levels	General features
Intensity.histogram.coefficient.of.variation	Intensity histogram features
X90th.discretised.intensity.percentile	Intensity histogram features
Intensity.mean.value	Intensity-based statistical features
Max.value	Intensity-based statistical features
Number.of.compartments..GMM.	Intensity-based statistical features
Intensity.based.interquartile.range..Original.Data.	Intensity-based statistical features (Original data)
Min.value..Original.Data.	Intensity-based statistical features (Original data)
Skewness..Original.Data.	Intensity-based statistical features (Original data)
Volume.at.intensity.fraction.10.	Intensity-volume histogram features
Volume.at.intensity.fraction.90.	Intensity-volume histogram features
Volume.fraction.difference.between.intensity.fractions	Intensity-volume histogram features
Global.intensity.peak	Local intensity features
Local.intensity.peak	Local intensity features
Cluster.shade	Grey level co-occurrence based features
Correlation	Grey level co-occurrence based features
Grey.level.variance..GLDZM.	Grey level distance zone based features
Large.distance.low.grey.level.emphasis	Grey level distance zone based features
Small.distance.emphasis	Grey level distance zone based features
Small.distance.high.grey.level.emphasis	Grey level distance zone based features
Small.distance.low.grey.level.emphasis	Grey level distance zone based features
Small.zone.emphasis	Grey level size zone based features
Zone.size.entropy	Grey level size zone based features
High.dependence.high.grey.level.emphasis	Neighbourhood grey tone difference based features
High.dependence.low.grey.level.emphasis	Neighbourhood grey tone difference based features

GMM, Gaussian mixture model; GLDZM, grey level distance zone matrix.
